# “The Four Realms of Existence. A New Theory of Being Human”

**DOI:** 10.17505/jpor.2026.29053

**Published:** 2026-03-26

**Authors:** Lars-Gunnar Lundh

**Affiliations:** Department of Psychology, Lund University, Sweden

**Keywords:** consciousness, cognition, mental models, anoesis, feeling, non-verbal expressions, music, embodiment, LeDoux

## Abstract

In *The Four Realms of Existence: A New Theory of Being Human*, Joseph LeDoux (2023) argues for a conceptualization of being human in terms of four hierarchically integrated realms of existence: biological, neurobiological, cognitive, and conscious. Although LeDoux’s perspective is thought-provoking, it also raises several questions. One question is how to differentiate the conscious realm from the cognitive realm. Another question is where feelings and non-verbal expressions fit into LeDoux’s picture. An overarching question is also: why *four* realms of existence?

The neuroscientist Joseph LeDoux, professor of science at New York University, is most known for his research on how neural circuits in the brain respond to threats, and how memories about threatening events are formed through cellular, synaptic and molecular changes in the amygdala. He has also authored several books, of which the latest is *The Four Realms of Existence: A New Theory of Being Human* (LeDoux, 2023). Here LeDoux asks the big question “What is a human being?” and argues that our understanding of this “has not advanced significantly beyond traditional ideas, some put forth in ancient times (LeDoux, 2023, p. 10). He also sees a stagnation in the treatment of mental disorders: “[a]ttempts to deliver treatments that were substantially better than those discovered in the 1950s and 1960s failed over and over” (p. 11). The reasons for this stagnation, he suggests, is not primarily due to technological limitations but more to a “lack of a rigorous, scientifically based understanding of what a human being is” (p. 11). He therefore argues for a radical rethinking of these matters, along the following lines:

I believe that a human being can be characterized as a composite of four fundamental, parallel, entwined realms of existence that reflect our evolutionary past and account for our present ways of being. We exist within these four realms of existence – biological, neurobiological, cognitive, and conscious – in every moment of life (especially adult life). But the kind of existence contributed by each realm is different. All four are, deep down, biological. But the neurobiological realm transcends the biological, the cognitive transcends the neurobiological, and the conscious transcends the cognitive. (LeDoux, 2023, p. 12).

LeDoux describes four hierarchically integrated realms of existence, where the basic *biological* realm is characterized by *life*-sustaining processes such as metabolism and the *species*-sustaining capacity for replication. Most living organisms have only biological existence. Animals, however, differ from other living things by also having a nervous system; they therefore exist also in a *neurobiological* realm. Some animals, in addition, exist in a cognitive realm, which means that they have

the ability to create internal representations of the environment and use these to construct mental models that make predictions about the world – all of which endow those animals with greater flexibility in responding behaviorally to challenges and opportunities in life. (LeDoux, 2023, p. 42)

Finally, some of these animals (at least humans) also exist in a conscious realm.

A partly similar approach to the understanding of human being has been presented by Damasio (2021), who speaks about *being*, *feeling*, and *knowing* as “three distinct and consecutive evolutionary stages” which correspond to “separable anatomical and functional systems that coexist in each of us humans” (Damasio, 2021, p. 25). In Damasio’s picture, *being* is something we share with all living organisms in the form of homeostatic and other biological processes, whereas *feeling* is found only in organisms with nervous systems, and *knowing* involves the development of consciousness and the expansion of the mind in all kinds of directions (for a review, see Lundh, 2025).

Another partly similar approach to the understanding of consciousness from an evolutionary perspective is that of Ginsburg and Jablonka (2019), who see the emergence of conscious beings as involving “the generation of a new functional realm, altering the very noting of living”. This makes it of interest to discuss similarities and differences between LeDoux’s approach and those of Damasio (2021) and Ginsburg and Jablonka (2019).

## Biological and Neurobiological

The defining features of the biological realm, as LeDoux describes it, are *metabolism* and *replication*. The biological realm developed around 3,7 billion years ago. Although it is unclear what developed first – metabolism or replication – it seems clear that once biological cells had developed,

the driving force that allowed each individual cell to persist (to live) was metabolism, while replication was required for the species to persist beyond the lifespan of the individual cell. (LeDoux, 2023, p. 56)

A central concept here is *homeostasis*. Homeostatic mechanisms work by continuously making various adjustments of the chemical milieu inside the body, stabilizing it in a way suitable for metabolism. Of particular importance is that each cell in the body can produce its own energy by using oxygen to break down glucose. Among other things, this requires that the fluids surrounding the cells must have a suitable concentration supply of oxygen and glucose; the chemical reaction that breaks glucose down also requires that the temperature is kept within a certain range. Without homeostatic mechanisms that regulate these processes, there would be no energy to keep the organism alive, nor to replicate.

Around 800 million years ago, the first animals with a nervous system appeared. As described by LeDoux, this made them capable of much *faster* responding, as compared with organisms that relied merely on the diffusion of chemicals between cells. Two innovations accounted for this: (1) the development of nerve cells (neurons) with axons that made cells able to interact over longer distances; and (2) the use of electrical signals that were much faster than the diffusion of chemicals.

The first nervous systems to develop were relatively simple nerve nets, consisting of a collection of interconnected neurons diffusely distributed throughout the living organism’s skin, such as are found today in jellyfish. Such nerve nets make possible various forms of sensory-motor integration that can serve the organism in its search for food and defense from predators. But, as LeDoux points out, it is important

to note that primitive animals like jellyfish don’t actually “perceive” the food or the predator as such. They have sensors that automatically respond to chemicals in nutrients by initiating an approach towards the food. And anything that touches their skin triggers an explosive burst of muscle contractions that propel escape. (LeDoux, 2023, p. 85)

What is at stake here, in other words, are simple stimulus-response associations.

An interesting aspect of evolution here is the development of organisms with new forms of embodiment, and the related developments of inner neural structures. Jellyfish belongs to the phylum *Cnideria*, which are *radial* in shape, possessing a single axis, a top and bottom, while having neither a front-back nor a left-right differentiation. An important development seems to have occurred when organisms with *bilateral bodies* developed around 630 million years ago. Flatworms are early examples of animals with bilaterally symmetrical bodies. And bilateral bodies, as LeDoux writes, “needed more than a nerve net” – they needed a centralized brain:

For a radial-bodied jellyfish, rapid and widespread muscle contraction, and the resulting undirected escape response, are enough for survival. But directional control over a bilateral body composed of three axes, and that can turn left when danger appears on the right, or can twist right when food is detected there, is only possible if there is some means to control diverse, spatially separated muscles in response to multiple kinds of stimuli. And that is what centralization provided. (LeDoux, 2023, p. 86-87)

As I see it, this provides a beautiful example of how changes in *embodiment* (from a radial body to a bilateral body) can have played an important role in the development of the nervous system. I was therefore somewhat confused when reading what LeDoux has to say about embodiment in another passage:

for me, the mind and brain are *not* embodied. Instead, the body is ‘embrained’… the body is represented in the brain via the capacities of the neurobiological realm. The neurobiological representation of the body is then re-represented in the mind via the processes of the cognitive realm, and some of these cognitive representations of the body are (or can be) subjectively experienced via the conscious realm. That’s how cognition and the body interact. (LeDoux, 2023, p. 45)

This exemplifies one problem I have with LeDoux’s theory. It is as if the only role he is willing to attribute to the body is as an inner *representation* of the body *in the brain* – despite his actual examples of important bodily changes during evolution, which show otherwise.

The development from nerve nets to brains in early bilateral animals led to the development of more complex forms of behaviour, such as fixed action patterns. Although fixed action patterns are much more complex than reflexes, they are still – like reflexes – automatically elicited by particular stimuli. As LeDoux points out, however, they are also influenced by *individual experience*, which is an important development. The importance of individual experience is also seen in the development of simple forms of learning, such as habituation, sensitization, conditioning, and the learning of habits by means of trial and error. All this, however, is described by LeDoux in neurobiological terms, which makes me wonder why there is a need to distinguish the neurobiological realm from the biological realm. Are they really to be seen as *two distinct realms of existence?*

It is interesting to compare LeDoux’s picture of the evolutionary importance of nervous systems with that described by Damasio (2021). In Damasio’s picture, the evolution of multicellular organisms with nervous systems led to the development of animals with *feelings*, and with a *perspective* on their surroundings. The nervous system, in Damasio’s description, made it possible for living beings to *feel the homeostatic processes in their body*, and thereby to regulate these in a more efficient way. This, according to Damasio, took the form of primordial feelings such as hunger, thirst, pain and pleasure, tiredness and alertness.

Feelings, however, have no role to play in LeDoux’s picture of the neurobiological realm. In his chapter on “viscerology” he describes how different visceral activities are controlled by neural circuits in the limbic forebrain, hypothalamus and brainstem, and how these “participate in processing loops between visceral tissues in the body and the various levels of the CNS” (LeDoux, 2023, p. 116). In principle, he describes similar kinds of interactions between body chemistry and the bioelectrical activity of neurons as Damasio does, but without using any words referring to feelings (e.g., of hunger and thirst). Here I wonder: Why should the neurobiological realm be differentiated as *separate from the biological realm* if it does not bring with it some kind of evolutionary *new* phenomena, such as feelings?

The reason for LeDoux’s unwillingness to use words that refer to feelings in this context seems to be epistemological. How do we know that an animal has feelings? This raises the question: What counts as *evidence* of feelings? I will return to this question in a separate section (“Where do feelings belong?) later in this review.

## The Cognitive Realm

With his conceptualization of the cognitive realm, LeDoux introduces a new level of description above the physiological level. Importantly, however, the *cognitive* realm, as he conceptualizes it, does not involve feelings or other subjective experiences – these belong to the *conscious* realm.

Central concepts in his description of the cognitive realm are *mental representations* and *mental models*. As he puts it, his criterion for distinguishing the cognitive realm from the neurobiological realm is “the ability to use the internal representation of information to construct mental models of the world” (p. 138). At the same time, this means that it is more difficult to decide which organisms are cognitive beings than to determine which are neurobiological beings:

Since there are no physical properties that can be called on to decide which animals exist cognitively, the workings of the cognitive mode of existence must be inferred from other properties, most typically from behavior. (LeDoux, 2023, p. 137).

As cognitive beings, we are able to use mental models, in the sense that we can “imagine novel solutions to problems without first having to test those via real-world actions” (p. 159). This *model-based cognition,* as LeDoux calls it, is found in mammals and at least some birds (e.g., corvids), but does not seem to exist in fish, reptiles and amphibians. As he summarizes the research in this area,

it is well established that mammals, when they pursue goals, can use internal representations to construct mental models that simulate the outcome of possible future actions (LeDoux, 2023, p. 166).

Why then did these kinds of cognitive capacities develop specifically in mammals and birds? Here LeDoux presents an embodiment-related hypothesis (again, however, without using the word embodiment), according to which both the position of the legs in mammals and their warm bloodedness (endothermy) were important for the evolution of model-based cognition. Crucial importance is attached to the fact that mammals had

legs positioned directly underneath their trunks that could move parallel to their vertebral column. As a result, they could breathe and run at the same time when escaping predators or capturing prey. Because this behavioral adaptation required more energy… [they] had to consume more food, but also had to take in more oxygen to make the extra energy, compared to almost all other groups of animals… The heat underlying these higher metabolic rates meant that mammals and birds were warm-blooded; that is, capable of maintaining their internal body temperature through metabolism. And warm bloodedness, or endothermy, may be the key to understanding the evolution of model-based cognition in mammals and birds. Specifically model-based cognition may have evolved as a way to obtain the fuel required for their high metabolic rates. (LeDoux, 2023, p. 171-172)

This suggests a picture of how, during evolution, the development of new forms of embodiment went hand in hand with development of new mental capacities. In this case, the capacity for model-based cognition made the animal able to “make plan about when (time of year and time of day) and where (locally and distally) to forage” (p. 172) for food.

LeDoux differentiates between *model-free* and *model-based* foraging. Model-free foraging belongs to the neurobiological realm and makes use of area-restricted search and learned habits, whereas model-based foraging makes use of internal representations to guide goal-directed behaviour. Both strategies use spatial maps (which involve place cells and grid cells in the hippocampus) for navigation. The distinguishing characteristic of *model-based* foraging is that “these spatial maps are part of a mental simulation of the possible outcomes” (p. 187).

This development of model-based cognition is said to involve a “prefrontal revolution”, with an increase in both the size and complexity of their neocortex. Special importance is ascribed to the increasing interconnectivity between different areas of the prefrontal cortex (PFC), such as the lateral PFC and the frontal pole. The lateral PFC is described as a *super-convergence zone* that not only integrates sensory information from different modalities with memory information, but also connects back to these input areas in a way that enables top-down control, and thereby serves as crucial substrate for processes in working memory:

The lateral PFC integrates sensory information not only within a single modality, but also between modalities… It is therefore referred to as a convergence zone that can form modality-independent representations… But the lateral PFC also receives input from other convergence zones in the temporal and parietal lobes, allowing even more abstract knowledge about the world, especially areas involved in memory… Because of its input from multimodal convergence zones, the lateral PFC is thought of as a *super-convergence zone*. Lateral PFC areas also connect back to their input areas, allowing top-down control of information processing in sensory and memory systems… lateral PFC can be thought of as a crucial substrate of both temporary storage and executive functions of working memory (LeDoux, 2023, p. 192-193)

In the final section of this part of the book, LeDoux summarizes the neural basis of human cognition in nine brief points, and argues that:

These processes, including processing within the mental model, I assume, can take place non-consciously. When that is the case, the *mere cognitive realm* is engaged. (LeDoux, 2023, p. 203)

This implies that a very large part of human cognition can occur non-consciously. This includes goal-directed behaviour, planning, memory search, deliberation, multi-tasking, conversation, etc. For example, LeDoux describes the verbal stream as largely separate from consciousness:

Words typically come out as sentences without you choosing each individual word and its grammatical placement – sometimes you do, but that is the exception… Verbal expressions and conscious experience are, in my scheme, separate, that is, parallel outputs of a non-conscious mental model and its non-conscious narrative (LeDoux, 2023, p. 287-288).

If so much cognitive and verbal activity can go on non-consciously, this naturally raises the question: What kind of mental processes *do* require consciousness?

LeDoux’s answer basically seems to imply that, so long as behaviour, thinking, speaking, etc. flows on in accordance with existing non-conscious cognitive models, there is no need for conscious considerations. He exemplifies this with a typical conversation, where

non-conscious active schema serve as templates for thought and speech. As a result, you can converse back and forth without having to think consciously about exactly what you are saying… But if the topic veers, or you disagree with the other person, then you probably need to consciously consider where to take things (LeDoux, 2023, p. 155).

This example seems to imply that, when something *unexpected* occurs that disturbs the spontaneous flow (“the topic veers, or you disagree with the other person”), *conscious thinking* is called upon.^1^

This might suggest the idea that all kinds of model-based *learning* require consciousness. Such a hypothesis has been suggested by Ginsburg and Jablonka (2019), although in terms of *unlimited associative learning* (UAL) – as distinguished from limited associative learning (LAL, such as habituation, sensitization, and simple form of conditioning). According to their hypothesis, UAL involves learning about *novel* stimuli and *compound* stimuli and requires consciousness, whereas LAL does not. Moreover, according to their theory, it is the *learning* phase that requires consciousness, whereas the *learned* behaviours and mental acts do *not*. As they exemplify,

the *encoding* during the learning of a compound novel stimulus… requires consciousness, but the recall of composite percepts and action patterns is very often unconscious (Ginsburg & Jablonka, 2019, p. 381).

In LeDoux’s terms of model-based cognition, this might translate into a distinction between model-based *learning* (which requires consciousness) and other varieties of model-based *cognition* (e.g., recognition, recall) which do *not*.

However, LeDoux does not explicitly draw any such conclusion. In fact, *if* model-based learning requires consciousness, this would seem to have ramifications for his theory of consciousness as a separate realm that *builds on* but transcends cognition. *If* the cognitive realm is defined in terms of “the ability to use the internal representation of information to construct mental models of the world” (LeDoux, 2023, p. 138), and *if* this kind of learning requires consciousness, then consciousness would seem to be a *prerequisite* for the development of model-based cognition. In other words, consciousness would have to be there *first*, before the development of model-based cognition. Once the mental models are *in place*, *after* learning, it enables us to function cognitively at a *non-conscious* level. The *development* of these mental models, however, would seem to require consciousness.

## The Conscious Realm

It is one thing to ask *which mental processes require consciousness*. It is another thing to ask about *the nature of consciousness*. It is certainly *not* the case that we are conscious *only* when we encounter novelty or complexity, or when we are engaged in the kind of learning that results in new mental models. On the contrary, we have conscious experiences continuously as we are awake. What happens when we encounter some unexpected novelty or complexity is *not* that consciousness is *turned on* by some kind of “switch”. What happens is rather that this unexpected novelty or complexity *attracts* a stream of consciousness *that is already there,* although with another focus. Consciousness is not awakened but merely gets a new focus.

According to LeDoux, we need to distinguish between two broad kinds of consciousness. The first kind, which he calls *creature consciousness*, “refers to the condition of being alive, awake, and behaviorally responsive to environmental stimuli” (p. 218). This, however, is not the kind of consciousness that interests him here. This kind of consciousness is, he says, “a feature of the neurobiological realm” (p. 218) because “all animals with nervous systems are conscious in this way, unless they are in coma or have otherwise suffered severe brain damage” (p. 218).

The other kind of consciousness, the kind that is of primary interest to LeDoux in this book, he *calls mental state consciousness*:

*Mental state consciousness* refers to the capacity to experience the world and one’s relation to it, and it exists only in animals with conscious realms. It is defined by the content of what one experiences, with that content supplied by a variety of brain systems that process information non-consciously, such as sensory, motor, memory, and cognitive systems, among others. (LeDoux, 2023, p. 219-220)

This means that, in LeDoux’s view, *experience* is an essential characteristic of consciousness.

LeDoux does not, however, present any integrated perspective on the conscious realm. To me, it seems rather that he is searching in various directions for an understanding of the nature of consciousness. I will focus here on three of these directions: (1) the concept of consciousness as a higher-order state in Rosenthal’s (2005) sense; (2) the role of narration and “mentalese”; and (3) Tulving’s differentiation between noetic, autonoetic, and anoetic consciousness.

### Consciousness as a “higher-order state

LeDoux explicitly adheres to a version of the “higher-order theory”, which means that what we are conscious of are aspects of lower-order perceptual and mental states *that are represented in higher-order states*. The higher-order state, however,

is not itself consciously experienced; it is instead a pre-conscious re-representation/re-description that allows the first-order state to be experienced. (LeDoux, 2023, p. 212)

This raises the question of what is involved in conscious experience *beyond* model-based cognition, in view of LeDoux’s thesis that consciousness represents a new realm, that can be clearly differentiated from the cognitive realm. If what is at stake here is merely the *re-representation* of mental contents from perception, memory and other aspects of mental functioning, as LeDoux suggests, why can’t this be simply another aspect (a *meta-*cognitive aspect) of *model-based cognition*? In other words: *if* conscious experience is *only* a matter of re-representation of mental contents, would it then be necessary to posit the existence of a conscious realm separately from the cognitive realm?

As LeDoux describes it, “higher-order consciousness depends on sustained activity in a working memory mental model” (p. 236), and this activity is sustained by “reciprocal connections that form processing loops between brain areas” (p. 236). His hypothesis is that continuous propagation through such loops “may be necessary to keep the overall cognitive network active long enough for a conscious experience to result and persist” (p. 237-238). Although this may be *necessary*, however, he clearly admits that it is not *sufficient* for conscious experience to occur:

To be clear, my proposal that conscious experience involves a preconscious working-memory mental model does not mean that working memory and mental models are sufficient for a conscious experience to occur. (LeDoux, 2023, p. 238)

What, then, is needed in addition to working memory and mental models? LeDoux’s suggestion here is that the missing piece of the puzzle is narration in a “language of thought” called Mentalese.

### Narration and mentalese

On page 243 LeDoux poses the question “How does a working memory mental model spawn conscious experience?” (p. 243) and suggests the following answer:

The short answer is that the mental model generates a story line, a narration, that creates the content of our conscious experiences. (LeDoux, 2023, p. 243)

This narration is assumed to take place in a modality-independent neural code, referred to as Mentalese:

these narratives do not come in verbal or visual or any other kind of recognizable code. They take the form of modality-independent, or “a-modal”, neural code, something called mentalese, that not only supplies our conscious content but also controls our speech and action. (LeDoux, 2023, p. 283)

Here LeDoux describes conscious experience as the result of an interaction between two mental models: a *pre-conscious* mental model (which transforms various kinds of input into mentalese) and a *conscious* model (which involves a re-representation of the contents from the pre-conscious model). In LeDoux’s description, the pre-conscious model has several functions. Besides supplying the contents of our conscious experience, it also enables us to engage in verbal and non-verbal expressive behaviours, and goal-directed acts. As he describes it, the preconscious model

allows its conceptual content to be used by diverse downstream processors. For example, it allows one to respond verbally through speech, writing, or sign-language, or non-verbally through a variety of distinct goal-directed actions…But the mentalese narrative also supplies the content of conscious experience.

The abstract mentalese narrative can, in other words, be thought of as a mental stream with three broad distributaries, or sub-streams, that diverge from the pre-conscious mental model. One is the *distributary of verbal expression*. It flows to cortical language circuits, making possible external linguistic communication about the contents of the narrative, The second is the *distributary of action*. It controls goal-directed behavior by way of connections with the basal ganglia and cortical motor outputs. And the third stream is the *distributary of consciousness,* the ground-zero of explicit conscious experiences. Unlike the two other streams, the *distributary of consciousness* remains within the confines of PFC working memory, where it populates a second mental model, a conscious one, with explicit content in the form of a conscious higher-order state. (LeDoux, 2023, p. 295)

Still, I wonder what makes one of these two mental models *conscious*, whereas the other remains *pre-conscious*. Don’t all mental models, by definition, belong to the *cognitive* realm? And how can the a-modal medium of Mentalese, which is *non-conscious* (as repeatedly stated by LeDoux) “spawn conscious experience” (p. 243).

### Noetic, autonoetic, and anoetic consciousness

The Canadian memory researcher Endel Tulving is best known for his differentiation between semantic memory and episodic memory – two different forms of explicit memory, as contrasted with implicit forms of memory (e.g., procedural memory). But Tulving (1985, 1993) also formulated a related distinction between three different kinds of consciousness: *noetic* consciousness (associated with semantic memory), *autonoetic* consciousness (associated with episodic memory) and *anoetic* consciousness (associated with implicit procedural memory).

Interestingly, autonoetic consciousness is described by LeDoux as “a very sophisticated form of mental modeling” (LeDoux, 2023, p. 246). Again, this raises the question about the relationship between mental modeling (which, according to LeDoux, belongs to the *cognitive* realm) and autonoetic consciousness (which is part of the *conscious* realm). As noted above, LeDoux maintains that mental models are *not sufficient* for conscious experience to occur (p. 238). This should therefore probably be the case also with autonoetic consciousness: that is, this kind of consciousness cannot be *reduced to* a sophisticated form of mental modeling. It requires something more, but what?

In line with Tulving’s conceptualization, one possible answer here is that conscious experience, *whether it is noetic or autonoetic*, also involves aspects of *anoetic* consciousness (*anoesis*, for short). As LeDoux puts it in one passage

anoesis is what makes noetic and autonoetic conscious states feel like something in us (LeDoux, 2023, p. 290).

This suggests that *feeling* is what makes the difference. Maybe conscious experience involves felt qualities that cannot be reduced to cognition, and that this is what is specific for the conscious realm? Examples of felt qualities are *familiarity* and *ownership*:

For example, when you enter your home, you don’t have to remind yourself that it is your home. You know it is your home because your brain has accumulated knowledge about it from you living there, and you are well acquainted with its features. Similarly, via lifelong mere acquaintance with your biological home (your body) and your psychological home (your mind), you are familiar with what your body and mental states feel like. The importance of this tacit feeling of ownership is most apparent when, through brain damage, it is eliminated. (LeDoux, 2023, p. 225)

In this context LeDoux introduces William James (1890) concept of the *fringe* of consciousness. As Mangan (2007) describes the fringe of consciousness, it consists of “feelings of relation”, without sensory content, which

make up the connective tissue which binds the relatively clear contents of consciousness together into larger wholes, and thereby constitute, among other things, our sense of temporality, continuity, meaning, and context (Mangan, 2007, p. 675).

Simple examples are when we search our memory for the name of a person, or for a word that might express something we want to say. As we try one name after another, or one word after another to fit the meaning we want to convey, we immediately have a feeling of whether it is the *right* or *wrong* name/word. These feelings of “right” or “wrong” illustrate what is meant by *anoetic* consciousness. LeDoux adds several other examples:

To Mangan’s list, I would add Leon Festinger’s notion of *cognitive dissonance*, which results in a feeling of “wrongness” when inconsistent beliefs sit side by side in the mind, and Daniel Oppenheimer’s notion of *fluency*, the vague subjective experience of ease or difficulty associated with completing a mental task. (LeDoux, 2023, p. 255)

Feelings of familiarity, ownership, right, wrong, cognitive dissonance, and fluency represent *anoetic* aspects of conscious experience. At the same time, they “point” to huge amounts of information that is there *implicitly,* in condensed form. As Mangan puts it,

the function of the fringe is to represent huge amounts of non-conscious context information in consciousness in *radically summarized or condensed* form… At any given moment, far more detailed information is potentially accessible *to* consciousness than, in fact, is actually *in* consciousness. This is the trick that lets consciousness finesse its otherwise severely limited capacity to represent information. (Mangan, 2003, p. 742-743)

Conscious attention is known to have a limited capacity. The fringe of consciousness serves as a complement to this limited capacity by its ability to represent, in condensed form, vast amounts of non-conscious information that are relevant to a given situation. According to Mangan, this is an adaptation that has been selected during evolution.

LeDoux develops a partly similar theme by arranging anoetic, noetic, and autonoetic consciousness in terms of their degree of energy consumption:

The default condition for consciousness is low-energy tacit anoesis, which uses procedural meta-cognition to implicitly conceptualize lower-order states and maintain fringe experiences in the conscious mental model. Higher-energy noetic consciousness is called on when explicit factual or conceptual content is needed to explicitly categorize and conceptualize external and internal events, but also to verify anoetic feelings of rightness, or cope with feelings of wrongness. Super-high-energy autonoetic consciousness is engaged when, and only when, episodic memory is deployed to explicitly conceptualize your personal involvement in a situation (LeDoux, 2023, p. 298).

All this seems to suggest that, after all, felt experience (feeling) has an important role in LeDoux’s scheme. But where?

## Where Do Feelings Belong?

Where, in LeDoux’s scheme, do *feelings* fit in? The obvious answer seems to be that they belong to the conscious realm. In some passages, he clearly argues that feelings such as fear are the result of cognitive interpretations that involve episodic or semantic memory:

The feeling of fear, in my view, is an autonoetic experience that results from the cognitive interpretation that you are in a situation of danger based on the presence of a stimulus that you have, from semantic or episodic experience, come to know of as dangerous. (LeDoux, 2023, p. 264)

One implication of this is that animals and infants cannot feel fear. In LeDoux’s perspective, without language we can feel *aroused* but not feel *fear*. To feel fear we must have the word fear in our vocabulary:

Emotion words categorize emotional experiences and provide conceptual anchors that help us understand and remember our experiences. These labels are not required in order to feel emotionally aroused, but they are required to feel the emotion named by the label. (LeDoux, 2023, p. 264)

Importantly, this implies that the *feeling* is there already before it is *labeled* as fear. It is there in the form of a felt *arousal*. But it is not fear until it is labeled as fear, and even adults may have difficulties in labeling a feeling as fear:

One may feel uncomfortable, concerned, or distressed in a situation, and not progress to something specific. But as the situation unfolds and more information is collected, it is also possible that a vague feeling may turn into one labeled and experienced as fear. (LeDoux, 2023, p. 265)

This seems to imply that we can have *vague* feelings even without labeling these. LeDoux clearly states that fear is a *specific* feeling that cannot be felt until we label it as fear. But how far can this position be generalized to other feelings?

Can’t we feel angry unless we label the feeling as anger?

Can’t we feel joy unless we label the feeling as joy?

Can’t we feel hungry unless we label the feeling as hunger?

Can’t we feel pain unless we label the feeling as pain?

Can’t we feel tired unless we label the feeling as tiredness?

One possibility here would be to distinguish between *sentience* and *consciousness*. Sentience has been defined as

*minimal animal consciousness* – the ability to have subjective experiences – rather than the ability to reflect about those subjective experiences, which seems to be a peculiar gift and curse of humans. (Ginsburg & Jablonka, 2019, p. 7)

As defined in this way, sentience only requires feelings to be *felt*, not to be *labeled*. Alternatively, this distinction might be formulated in terms of different *levels* of consciousness. LeDoux speculates along these lines on p. 276, where he suggests the possibility (1) that all mammals have “anoetic fringe conscious feelings”, (2) that anthropoid primates in addition have “noetic factual and conceptual consciousness”, and (3) that only humans and possibly some other great apes also have the capacity for “autonoetic consciousness”. He suggests the possibility that there may exist

a primitive – perhaps first-order – kind of state (sentience)… between anoesis and creature consciousness (LeDoux, 2023, p. 280-281).

Anyway, this suggests the importance of differentiating between “having a feeling” and “knowing a feeling” (e.g., Damasio, 2000, p. 284)

This raises the question: What counts as *evidence* of having feelings? LeDoux speaks of two kinds of evidence of consciousness: verbal self-reports and neural similarities (pp. 194–202, 268–281). But what about *non-verbal expressions*? An important thing about feelings is that they have non-verbal expressions, which are intersubjectively perceivable and thereby amount to a kind of “body language”.

According to LeDoux, verbal report is the “most straightforward and reliable way of distinguishing mental state consciousness from non-conscious processes” (p. 269); it is described as “the gold standard in assessing consciousness in humans” (p. 269). But does this also apply to feelings? If verbal and non-verbal evidence point in different directions, do we always choose to rely on the verbal evidence? Consider the case of someone who exclaims “I am not angry”, with an angry voice. What do we primarily tend to believe here: the verbal message, or the non-verbal expression? I guess most of us, in most such cases, would regard the non-verbal expression as more reliable. At the very least, this kind of example demonstrates that we do not *always* regard verbal expressions as more reliable than non-verbal expressions as evidence of a person’s feelings.

It has been argued that non-verbal expressions are *a-modal,* at least to a certain extent. For example, the same kind of bodily expression can both be *felt* in oneself and *seen* in others. Such non-verbal expressions thereby

enable an *intersubjective bridge* between individuals, as the same expressions that we recognize in ourselves are typically also perceivable in others. (Lundh & Foster, 2025, p. 11)

Some basic examples are laughter and yawning, which can spread from one person to another. More generally, *emotional contagion* involves a spontaneous spread of emotions that leads to synchronization between individuals.

The intersubjectivity of non-verbal expressions is also seen in human practices where feelings are *intentionally communicated*. In dancing performances, both the dancers and the audience may experience the same kind of emotions. As exemplified by Forlé (2024, p. 565), a tango “can express passionate love even though the dancers barely know each other and are definitely not in love with one another.”

The same goes for acoustic communication. Verbal speech is accompanied by *prosodic* features (e.g., intonation, stress rhythm, loudness) that reflect the speaker’s feelings and make these feelings perceptually available to others. Research demonstrates that the acoustical patterns in music show similarities to the vocal expressions used to communicate basic emotions (Juslin & Laukka, 2003). As Juslin (2013) puts it, listeners perceive *emotional meaning* in music, which is a reason why “music is sometimes, perhaps justifiably so, called a universal language of the emotions” (p, 11).

Listening to music and watching dancing performances, like the perception of non-verbal expressions of feelings in everyday interaction with others, clearly illustrate conscious experiences that are *not verbally mediated*. A sustainable theory of consciousness must take account of these kinds of experiences. Here it is hardly the case that verbal reports count as “the gold standard in assessing consciousness”.

LeDoux’s apparent over-reliance on verbal evidence of conscious experiences also strikes me as somewhat at odds with his own model of the human mind, where he describes the verbal stream as largely *separate* from consciousness:

Verbal expressions and conscious experience are, in my scheme, separate, that is, parallel outputs of a non-conscious mental model and its non-conscious narrative. (LeDoux, 2023, p. 288)

It would have been interesting to see a more detailed analysis of what this *separateness* of the verbal stream from the conscious stream implies for the reliability and fallibility of verbal reports as evidence of mental processes.

The question to what extent *non-verbal* expressions can be relied on as evidence of feelings is perhaps analogous to the question to what extent we can rely on *verbal* expressions as evidence of conscious experiences. In both cases, we have an almost irresistible tendency to perceive such expressions as evidence of feelings, thoughts, intentions, etc. But in neither case is this *evidence* equivalent to *proof* – that is, in both cases, we may be deceived by the appearances. Like scientific hypotheses, both kinds of perceptions are *fallible*.

Consider the classical experiment by Heider and Simmel (1944), where they presented observers with simple animated geometric shapes that moved around on a screen, seemingly on their own accord (i.e., without being pushed into action by some external cause). The results showed that the observers tended to perceive these geometric shapes as characters with feelings, intentions, and other experiences. This illustrates how certain patterns of movement tend to be directly perceived as *expressive* of feelings and intentions. Even though we do not confuse such geometric shapes with real human beings, we have an almost irresistible tendency to experience their movements as expressive of feelings and intentions.

It seems that we have a similar tendency to experience AI-based language models as real individuals that are expressing their thoughts. Jonathan Birch refers to this as *the persisting interlocutor illusion*:

At present, many users seem to misunderstand the true nature of their interactions with chatbots in significant ways. Chatbots generate a powerful illusion of a companion, assistant, or partner being present throughout a conversation. I call this *the persisting interlocutor illusion*. (Birch, 2025, p. 4)

This illustrates how verbal expressions are fallible in a similar way to non-verbal expressions.

## Why *Four* Realms of Existence

LeDoux may very well be right when he argues that the present stagnation in the understanding and treatment of mental disorders is primarily due to a “lack of a rigorous, scientifically based understanding of what a human being is” (LeDoux, 2023, p. 11). His book is a thought-provoking work that raises many questions. I am skeptical, however, about his notion of four realms of existence. Why *four*?

First, why not include also a *physical* realm? Are we not also physical creatures, in addition to being conscious, cognitive, and biological creatures? Second, why differentiate between a biological and a neurobiological realm – isn’t the neurobiological a part of the biological realm? And third, on what grounds do we have to posit a separate *cognitive* realm? As LeDoux points out, cognitive phenomena unlike biological and neurobiological phenomena are *not observable*: “the workings of the cognitive mode of existence must be inferred from other properties, most typically from behavior” (p.137). And, as he also emphasizes, the cognitive realm is *not experienced*; in other words, it is assumed to be entirely *non-conscious*. If it can neither be observed from a third-person perspective, nor be experienced from a first-person perspective, why should it be given the status of a realm of *existence*?

Although I am skeptical about the notion of *four* realms of existence, the conceptualization of consciousness as a *realm* is worth further exploration. Ginsburg and Jablonka (2019) express a partly similar idea when they argue that consciousness (in analogy with life) represents the development of *a new functional realm*. They also provide a reason why it may be a good idea to speak about consciousness as an entire *realm,* when they claim that “the question about the function of consciousness is misleading” (p. 185):

Just as it would be a category mistake to ask what the functions of being alive are, so it is a category mistake to ask what the functions of being conscious include. (Ginsburg & Jablonka, 2019, p. 188)

In Ginsburg and Jablonka’s view, consciousness represents “a new functional realm”, *in analogy with the development of life*. Just as it is misplaced to ask about the function of life, it is also mistaken to ask for the function of consciousness. Both life and consciousness represent whole systems (teleological systems) with intrinsic *goals*.

it does make a lot of sense to ask about the intrinsic goal of living or the intrinsic goal of consciousness, for goals can be attributed to whole systems, while biological functions are defined as the parts and processes that contribute to the operation of a goal-directed encompassing system. The emergence of conscious beings led to the generation of a new functional realm, altering the very noting of living. (Ginsburg & Jablonka, 2019, p. 188)

Whereas the intrinsic goals of all living individuals can be defined as survival and reproduction, they see the evolution of consciousness as involving the development of a new causal force of “intrinsic motivating subjective experience” (p. 188), on top of the more basic physical and biological forms of causality. This new form of causality is said to require a new explanatory framework, which they describe in terms of “felt needs” and the ascription of value to complex stimuli and actions (Ginsburg & Jablonka, 2019, p. 480).

Living became a goal in a new way, a highly individualized way, a conscious way, and this can explain the evolution of multiple cognitive and affective functions. (Ginsburg & Jablonka, 2019, p. 189).

Despite their apparent differences (e.g., Ginsburg and Jablonka place the development of consciousness at an earlier stage in evolution than LeDoux does), both LeDoux and Ginsburg & Jablonka point in interesting ways to an understanding of consciousness as an encompassing *realm*. From this kind of perspective, we should ask not only about the types of *contents* of consciousness (e.g., perceptions, memories, thoughts, feelings, intentions, motives, etc.) but also about the *structure* of conscious experience (e.g., focus/fringe, figure/background, implicit horizons, etc.), and its *dynamics* (e.g., ever changing perspectives due to the individual’s orientation in the surroundings, spontaneous conscious activity, and selective attention).

One question here is if the conscious realm should be defined as including not only what we are conscious of at a specific *moment*, but also what we can become conscious of by a turn of attention. If so, the conscious realm would include all kinds of *preconscious* contents – that is, perceptions, memories, thoughts, feelings, etc. that we have conscious *access* to. These *pre*conscious contents would have to be clearly differentiated from truly *non-conscious* processes (i.e., processes that we do not have access to). Maybe this would also mean that one part of the so-called cognitive realm (the *pr*econscious processes) could be integrated within the conscious realm, whereas the truly non-conscious processes could be placed in the biological realm.


*Lars-Gunnar Lundh*


## Footnotes

1 Cognitive psychologists have long spoken similarly of *automatic* versus *controlled* information processes (e.g., Shiffrin and Schneider, 1977). Au-tomatic information processing is fast, effortless, and unconscious, and is sufficient to handle of all kinds of well-practiced tasks. When novel or complex situations occur, however, this calls for controlled information processing, which involves intentionally initiated sequences of cognitive activities that require *conscious attention* and *effort*.

## Open access

This article is distributed under the terms of the Creative Commons Attribution 4.0 International License (http://creativecommons.org/licenses/by/4.0/), which permits unrestricted use, distribution, and reproduction in any medium, provided you give appropriate credit to the original author(s) and the source, provide a link to the Creative Commons license, and indicate if changes were made.
